# Side Effects of Yttrium-90 Radioembolization

**DOI:** 10.3389/fonc.2014.00198

**Published:** 2014-07-29

**Authors:** Ahsun Riaz, Rafia Awais, Riad Salem

**Affiliations:** ^1^Section of Interventional Radiology, Department of Radiology, Robert H. Lurie Comprehensive Cancer Center, Northwestern University, Chicago, IL, USA

**Keywords:** radioembolization, complications of cancer therapy, side effects, liver neoplasms, radiation effects

## Abstract

Limited therapeutic options are available for hepatic malignancies. Image guided targeted therapies have established their role in management of primary and secondary hepatic malignancies. Radioembolization with yttrium-90 (^90^Y) microspheres is safe and efficacious for treatment of hepatic malignancies. The tumoricidal effect of radioembolization is predominantly due to radioactivity and not ischemia. This article will present a comprehensive review of the side effects that have been associated with radioembolization using ^90^Y microspheres. Some of the described side effects are associated with all transarterial procedures. Side effects specific to radioembolization will also be discussed in detail. Methods to decrease the incidence of these potential side effects will also be discussed.

## Introduction

### Primary hepatic malignancies

Hepatocellular carcinoma (HCC) and intra-hepatic cholangiocarcinoma (ICC) are primary liver malignancies. HCC is much more common than ICC (1, 2). Surgical resection is reserved for a select group of patients with resectable disease (3). Orthotopic liver transplantation may be performed in patients with HCC who are within the Milan criteria (4). Chemoembolization and radiofrequency ablation are considered standard locoregional therapies for patients with unresectable HCC (5, 6). Radioembolization is an alternative locoregional therapy, which has established its role in the management of primary liver tumors.

### Secondary hepatic malignancies

Malignancies commonly metastasize to the liver (7). Hepatic metastases are generally managed by surgical resection or systemic medical treatments. Radioembolization for hepatic metastases is safe and effective in secondary hepatic malignancies (8–10).

### Radioembolic agents

^90^Y microspheres are used in treatment of hepatic malignancies. The details of ^90^Y are beyond the scope of this manuscript. Table 1 presents the relevant differences in the two available ^90^Y microsphere devices.

**Table 1:** **Yttrium-90 microsphere devices**.

Name	TheraSphere^®^	SIR-Spheres^®^
Material	Glass microsphere	Resin microsphere
Size of particle (microns)	20–30	20–60
Embolic effect	Mild	Mild to moderate
Doses	3–20 GBq	3 GBq
Number of particles per treatment	1.2–8 million	Up to 30 million

SIR-Spheres^®^ are FDA-PMA (Food and Drug Administration-Premarket Approval) approved for metastatic colorectal cancer to the liver (11). TheraSpheres^®^ are FDA approved under HDE (humanitarian device exemption) for radiation treatment or as neo-adjuvant to surgery or transplantation in patients with HCC who can have appropriately placed hepatic arterial catheters (12). This device is indicated for HCC patients with partial or branch portal vein thrombosis/occlusion when clinical evaluation warrants the treatment. Other investigational uses of these devices are being employed.

Multiple other radioactive devices are being investigated for transarterial therapy. These include iodine-131 labeled iodized oil, rhenium-188 HDD labeled iodized oil, phosphorus-32 glass microspheres, and Milican/holmium-166 microspheres.

### Pre-treatment assessment

Pre-treatment evaluation of radioembolization includes:

- Pre-treatment clinical evaluation
- Pre-treatment laboratory evaluation
- Pre-treatment radiological evaluation
- Pre-treatment angiography

#### Pre-treatment clinical evaluation

A multidisciplinary team consisting of hepatologists, medical/surgical/radiation oncologists, transplant surgeons, and interventional radiologists should select patients for radioembolization. A clinic visit is necessary. A history, which includes patient’s prior surgical and medical therapies, is necessary. A recent article suggested safety of radioembolization in patients who have had prior partial hepatectomies (13). The patient’s performance status per Eastern Cooperative Oncology Group (ECOG) should be assessed.

#### Pre-treatment laboratory evaluation

Appropriate laboratory tests including but not limited to liver function tests and corresponding tumor markers should be performed to ascertain baseline values. For patients with cirrhosis, it is essential to classify patients. The Child-Pugh classification is commonly employed by multiple disciplines and includes the following variables:

a) Serum bilirubin
b) Serum albumin
c) PT/INR
d) Encephalopathy
e) Ascites

#### Pre-treatment cross-sectional imaging evaluation

A triphasic liver CT or MRI is usually performed to evaluate the following:

a) Extent of disease
b) Location of disease
c) Relative tumor hypervascularity
d) Variant vascular anatomy

#### Pre-treatment angiography

**Figure 1:**
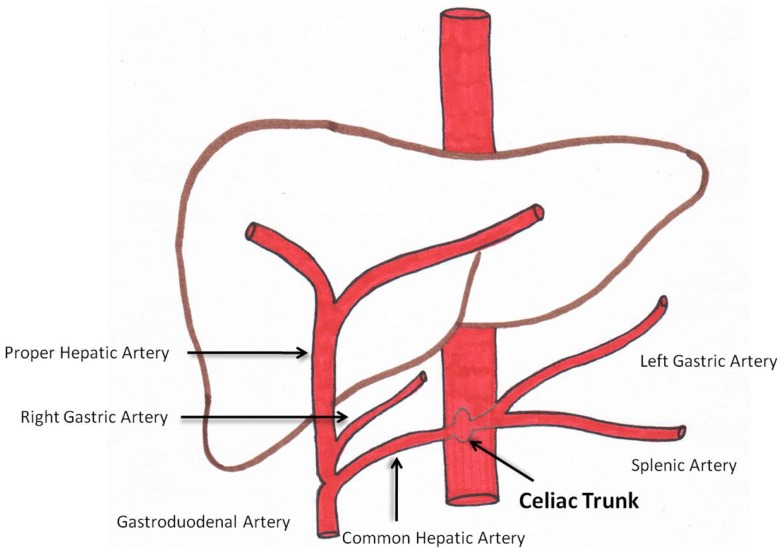
**Schematic representation of celiac arterial anatomy**.

Angiography prior to radioembolization is essential. This provides the interventional radiologist with knowledge of the hepatic arterial anatomy and aberrant vasculature (14). Figure 1 is a diagram representing conventional celiac arterial anatomy.

An aortogram assesses aortic atherosclerosis and tortuosity. A superior mesenteric angiogram is essential to determine variant vessels to the liver. Delayed images can assess the patency of the portal vein. A celiac angiogram determines hepatic vasculature and variants. Segment 1 tumors require special attention given various potential contributing feeders (15). More selective angiography with microcatheters/microwires is recommended to determine a safe and efficacious point of radioembolization to the tumor.

#### Coil embolization

Coil embolization of communicating vessels that may lead to aberrant microsphere deposition can be performed if necessary. The aberrant deposition of microspheres in the gastrointestinal tract (GIT) or pancreas can have grave consequences (16–18). Some vessels that may need to be coil embolized prior to treatment are: gastroduodenal artery (GDA), right gastric artery (RGA), accessory left gastric artery, falciform artery, phrenic arteries, inferior esophageal artery, supraduodenal artery, and retroduodenal artery.

Pre-treatment prophylactic coil embolization is dependent on the following variables (19, 20):

- Experience of the treating physician
- Planned location of radio-microsphere delivery
- Size of vessel

Collateral hepaticoenteric flow can develop following coil embolization. This may increase aberrant microsphere deposition on following repeat treatments. Theoretically, if the interval between coil embolization and radioembolization is long, this phenomenon can also occur during the initial treatment. Further research on this issue is needed.

### Potential methods to enhance/confirm tumor delivery of radiomicrospheres

#### C-arm CT

Appropriate tumor targeting is now routinely confirmed by using C-arm CT. This method aids in accurately recognizing non-tumor/non-hepatic contrast delivery.

#### Consolidation of hepatic arterial flow

Variant hepatic artery and parasitized hepatic artery can be coil embolized prior to radioembolization. This leads to intra-hepatic collateralization in preparation for radioembolization.

#### Angiotensin II

A systematic review concluded that Angiotensin II could increase tumor to non-tumor blood flow by approximately up to threefold (21). However, further studies are needed to determine systemic safety profile as Angiotensin II could increase systemic blood pressure.

#### Degradable starch microspheres

A five patient analysis on the use of degradable starch microspheres as an embolizate to normal hepatic parenchyma during radioembolization was performed. Post-radioembolization SPECT/CT demonstrated sparing of normal parenchyma (22). This is an interesting concept, which needs validation.

### Technetium-99m macroaggregated albumin (^99m^Tc-MAA) scan

**Figure 2:**
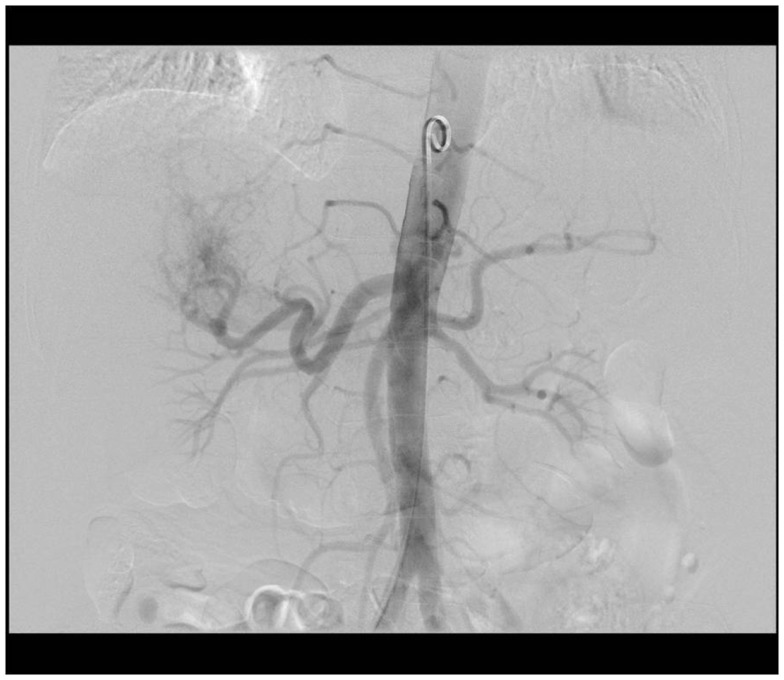
**Angiographic image demonstrating hypervascular tumor**.

**Figure 3:**
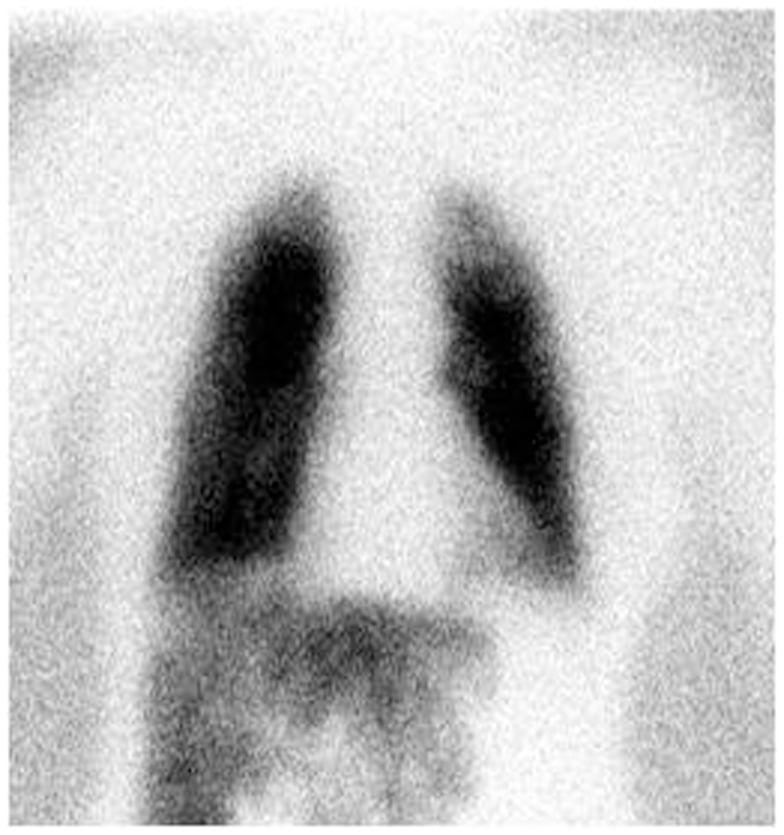
**Planar Tc-99m MAA scan demonstrating high LSF (76%)**.

A ^99m^Tc-MAA scan is performed to assess the lung shunt fraction (LSF) and splanchnic shunting. Figure 2 shows a hypervascular HCC. Figure 3 shows planar scintigraphic imaging from a nuclear medicine scan demonstrating significant LSF. SPECT can enhance detection of splanchnic flow. However, conventional angiography is considered standard for identifying GI uptake by most interventional radiologists (23).

In 2011, Sabet et al. published an article demonstrating that oral administration of sodium perchlorate before the test angiogram with ^99m^Tc-MAA resulted in effective avoidance of free ^99m^Tc-pertechnetate concentration and decreased incidence of equivocal findings in the gastroduodenal region (24).

### Dose calculations

A brief overview of dose calculations is important to understand potential complications. Recent data suggest that ^99m^Tc-MAA (simulates ^90^Y) and ^99m^Tc-sulfur colloid (SC) (accumulates in reticuloendothelial tissue), dual tracer SPECT/CT may provide more accurate dose calculations as this method provides a more accurate dose to functional liver (DFL) (25). Research is being done on the utilization of PET/CT for dosimetry (26).

#### Dose calculation for TheraSphere^®^

Dose is calculated for TheraSphere^®^ using the following formula:

$$\text{Dose}\;\left(\text{Gy}\right)=\frac{\text{50}\left[\text{Injected}\;\text{activity}\;\left(\text{GBq}\right)\right]\left[\text{1}-\text{LSF}\right]}{\text{Liver}\;\text{Mass}\;\left(\text{kg}\right)}$$

Per the TheraSphere^®^ package insert, the upper limit of injected activity to the lungs is 0.61 GBq (12).

#### Dose calculation for SIR-Sphere^®^

The two acceptable methods for individual patient dose calculation for SIR-Sphere^®^ include the partition model and empirical model (11). The empirical model is based on dose known from previously published clinical data and chooses the safest and most effective dose from it. The recommended patient dose is based on percent involvement by the tumor in the liver. A 3 GBq vial is used for greater than 50% hepatic tumor involvement; a 2.5 GBq vial is used for 25 to 50% hepatic tumor involvement; a 2 GBq vial is used for less than 25% hepatic tumor involvement. The package insert for SIR-sphere^®^ acknowledges that individual patient dose calculation is complex.

Additionally, LSF affects dose reduction. If there is less 10% LSF, there is no reduction in dose. If there is 10–15% LSF, the dose is reduced by a factor of 20%. If there is 15–20% LSF, the dose is reduced by a factor of 40%. If the LSF is greater than 20%, treatment is not recommended.

### Single-session radioembolization

A recently published method of single-session radioembolization (pre-treatment angiography/Tc-99m MAA scan/radioembolization on same day) employed by the group showed no reportable events. In this analysis, planar scintigraphy was performed in 2 h following administration of Tc-99m MAA and LSF was determined. Final dosimetry calculations were performed while the patient was being transferred back from nuclear medicine to interventional radiology. This method decreased the costs and time between initial clinical assessment and radioembolization (27). It should be noted that this method requires high level of expertise and efficient communication between the nuclear medicine department, physicist, and interventional radiologist.

### Post-treatment assessment

#### Post-treatment imaging

At our institution, cross-sectional contrast enhanced imaging (triphasic CT or MRI) is obtained at 1 month following treatment and at 3 month intervals following the first post-treatment imaging. This protocol evaluates response, or lack thereof, to treatment.

Bremsstrahlung SPECT/CT and PET/CT are being investigated for evaluating post-treatment technical success and predicting treatment efficacy (26). Time of flight PET/CT has improved spatial resolution when compared to Bremsstrahlung SPECT/CT. Aberrant microsphere deposition may also be identified using SPECT/CT and/or PET/CT. Gupta et al. have published a case report of aberrant delivery of ^90^Y to the duodenum identified on PET/CT performed after radioembolization (28). Early knowledge of aberrant microsphere deposition may lead to early interventions.

#### Post-treatment laboratory evaluation

Post-treatment LFTs and complete blood count are usually performed 1 month following treatment. Tumor markers (such as alpha-fetoprotein for HCC) may aid in assessing response to therapy.

## Complications of Radioembolization

The complications occurring after radioembolization (29) are discussed in detail below. Table 2 summarizes available data on post-radioembolization complications.

**Table 2:** **Summary of available data on post-radioembolization complications**.

Complications	Reference	Materials	Findings/conclusion(s)
Hepatic	Young et al. (30)	41 HCC patients with multiple treatments to same segment/lobe	Okuda I: can tolerate up to 390 Gy Okuda II: can tolerate up to 196 Gy
	Sangro et al. (31)	45 Patients with liver tumors	RILD increases with: increasing age, whole liver treatment, and elevated baseline bilirubin levels
	Kennedy et al. (32)	680 Liver tumor ^90^Y treatments with resin microspheres	RILD increases with: increased activity and use of the empiric method for dose calculation
Biliary	Atassi et al. (33)	327 Patients with liver tumors	Biliary necrosis (*n* = 17)
			Bilomas (*n* = 3)
			Cholecystitis (*n* = 2)
			Gall bladder wall rent (*n* = 3)
			Abscess (*n* = 1)
			Biliary strictures (*n* = 8)
	Ng et al. (34)	2 Biliary complications	Biliary stricture (*n* = 1)
			Cholangitis (*n* = 1)
Pulmonary	Leung et al. (35)	80 Patients with liver tumors	Radiation pneumonitis (*n* = 5; 6.3%).
			Pulmonary complications increase in patients with LSF > 13%
	Salem et al. (36)	403 Patients with liver tumors	Radiation pneumonitis (*n* = 0)
			Grade I toxicities per RTOG/EORTC^a^ criteria (*n* = 10; 19%)
Gastrointestinal	Carretero et al. (37)	78 Patients	Gastroduodenal injury (4%)
	Murthy et al. (38)	Patients with liver tumors	Important to recognize hepaticoenteric arterial communications
	Mallach et al. (39)	One case of gastroduodenal ulceration	Endoscopy is required to confirm
	Szyszko et al. (40)	21 Patients	GI ulceration in 29% patients
	South et al. (41)	27 Patients	GI ulceration in 11% patients
	Lam et al. (42)	247 Patients	GI ulceration in 3.2%

*^a^RTOG/EORTC, radiation therapy oncology group/European organization for research and treatment of cancer*.

Please note that it is important to standardize recording and reporting toxicities. Clinical and laboratory toxicities may be classified according to the standard criteria such as the Common Terminology Criteria for Adverse Events (CTCAE) version 4.0, whenever possible (43). Tables 3 and 4 give some of the relevant clinical and laboratory (investigational) toxicities according to CTCAE v4.0.

**Table 3:** **Some relevant clinical toxicities according to the CTCAE v4.0**.

Clinical toxicity	Grade				
	1	2	3	4	5
Diarrhea	Increase of <4 stools per day over baseline; mild increase in ostomy output compared to baseline	Increase of four to six stools per day over baseline; moderate increase in ostomy output compared to baseline	Increase of ≥7 stools per day over baseline; incontinence; hospitalization indicated; severe increase in ostomy output compared to baseline; limiting self care ADL	Life-threatening consequences; urgent intervention indicated	Death
Nausea	Loss of appetite without alteration in eating habits	Oral intake decreased without significant weight loss, dehydration or malnutrition	Inadequate oral caloric or fluid intake; tube feeding, TPN, or hospitalization indicated		
Pancreatitis	–	Enzyme elevation or radiologic findings only	Severe pain; vomiting; medical intervention indicated (e.g., analgesia, nutritional support)	Life-threatening consequences; urgent intervention indicated	Death
Vomiting	One to two episodes (separated by 5 min) in 24 h	Three to five episodes (separated by 5 min) in 24 h	≥6 episodes (separated by 5 min) in 24 h; tube feeding, TPN or hospitalization indicated	Life-threatening consequences; urgent intervention indicated	Death
Abdominal pain	Mild pain	Moderate pain; limiting instrumental ADL	Severe pain; limiting self care ADL	–	–

**Table 4:** **Some relevant laboratory toxicities according to the CTCAE v4.0**.

Laboratory toxicity	Grade				
	1	2	3	4	5
Bilirubin	ULN to increase of >1.5 × ULN	Increase of 1.5–2.5 × ULN	Increase of >2.5 × ULN	–	–
INR	ULN to increase of >1.5 × ULN; increase of >1–1.5 × baseline if on anticoagulation	Increase of 1.5–2.5 × ULN; increase of >1.5–2.5 × baseline if on anticoagulation	Increase of >2.5 × ULN; increase of >2.5 × baseline if on anticoagulation	–	–
Alanine aminotransferase	ULN to increase of >3 × ULN	Increase of 3–5 × ULN	Increase of 5–20 × ULN	Increase of >20 × ULN	–
Aspartate aminotransferase	ULN to increase of >3 × ULN	Increase of 3–5 × ULN	Increase of 5–20 × ULN	Increase of >20 × ULN	–
Alkaline phosphatase	ULN to increase of >2.5 × ULN	Increase of 2.5–5 × ULN	Increase of 5–20 × ULN	Increase of >20 × ULN	–
Lymphocyte count decrease	LLN to 800/mm^3^	500–800/mm^3^	200–500/mm^3^	<200/mm^3^	–
Platelet count decrease	LLN to 75,000/mm^3^	50,000–75,000/mm^3^	25,000–50,000/mm^3^	<25,000/mm^3^	–

*ULN, upper limit normal; LLN, lower limit normal*.

## Post-Radioembolization Syndrome

A post-radioembolization syndrome (PRS) includes fatigue, nausea/vomiting, abdominal pain/discomfort, and/or cachexia. PRS is less severe than that observed after embolic therapies. Hospitalization is rarely required (44–47). Incidence of PRS ranges from 20 to 70% (17, 44–46). In a two-institution, 112-patient analysis, the incidence of PRS was 70% (48). Patients should be made aware of these potential side effects before therapy. A 2-week post-radioembolization telephone call is recommended to inquire for symptoms of PRS. A clinic visit 1 month following treatment is recommended to clinically assess the patient.

### Nausea/vomiting

Nausea and vomiting may occur following radioembolization. Based on this experience, antinauseants/antiemetics such as ondansetron are routinely administered prior to treatment. Antinauseants/antiemetics pro re nata (PRN) are usually sufficient to treat nausea/vomiting following treatment.

### Pain

Patients may experience right upper quadrant pain and/or generalized abdominal discomfort. Over the counter analgesics, PRN usually treat the discomfort/pain following radioembolization. Stronger analgesics such as opiates are rarely necessary.

## Complications Due to Aberrant Microsphere Deposition or Radioactivity to Surrounding Structures

### Hepatic dysfunction

Pre-existing liver dysfunction is a significant confounding variable when assessing post-radioembolization liver toxicities in HCC patients (30). It is important to classify these cirrhotic patients according to their liver function prior to treatment as previously discussed. A Child-Pugh class C (score of greater or equal to 10) is usually considered a contraindication to locoregional therapies. Patients with elevated baseline bilirubin (>2 mg/dL) are generally not considered ideal candidates. As the background liver parenchyma is usually normal in patients with hepatic metastases, liver function tests are usually within normal limits. This may not be the case where a majority of the liver is replaced by tumor.

Radiation-induced liver disease (RILD) is a potentially serious post-radioembolization complication (31). Given complexity of radioembolization dosimetry, using the empirical method for dose calculation is not recommended when using SIR-Spheres^®^. The incidence of RILD after ^90^Y radioembolization ranges from 0 to 4% (30–32). RILD occurs due to the exposure of normal hepatic parenchyma to radiation. The embolic (ischemic) effect of these microscopic particles is minimal and is not thought to contribute to the hepatotoxicity from radioembolization.

#### Available data

A recent article demonstrated repeated radioembolization to be a significant risk factor in development of radioembolization induced liver disease (49). Two of the patients in their eight-patient analysis who had received multiple radioembolization treatments died with clinical features of RILD.

Prior exposure of the liver to external beam radiation therapy (EBRT) may lead to increased liver toxicity after radioembolization. This depends on fractional liver exposure and dose level. The authors concluded that radioembolization appears to be safe for the treatment of hepatic malignancies only in patients who have had limited hepatic exposure to prior EBRT (50).

As biochemical aberrations may occur without clinical manifestations, follow-up liver function tests are routinely recommended 1 month after treatment. In rare cases of clinically manifest RILD, a biopsy of the normal parenchyma may help confirm the diagnosis. A case of post-radioembolization fulminant hepatic failure has been reported (51).

In a single center article analyzing hepatic dysfunction following radioembolization with SIR-Spheres^®^, liver function toxicity (grades 1 through 4) was seen in 58% of infusions. The median duration of LFT toxicities was 20–29 days. Grade 3 or greater toxicities occurred after 9% of infusions in their analysis. One patient died in 32 days of treatment with signs and symptoms compatible with radiation-induced liver disease (52).

#### Dose to functional liver

^99m^Tc-SC SPECT has been used to calculate DFL. ^99m^Tc-SC accumulates in normal liver parenchyma due to presence of reticuloendothelial tissue. Increased post-radioembolization liver enzyme elevation was seen with increased DFL (25).

#### Hepatic fibrosis/portal hypertension

Pre-existing findings consistent with portal hypertension are not a contraindication to radioembolization. Post-radioembolizaiton hepatic fibrosis and/or portal hypertension are potential post-treatment complications (53). In an analysis by Jakobs et al. (54), 32 patients with secondary hepatic malignancies were selected to exclude the confounding variable of cirrhosis. Mean decrease in hepatic volume was 11.8% and mean increase in splenic volume was 27.9% in patients who had undergone bilobar radioembolization. The authors concluded that radioembolization may cause portal hypertension by imaging criteria. However, no patients exhibited any clinical sequelae of portal hypertension. Clinically significant manifestations such as reduced platelet counts (<100,000/dL) or variceal bleeding are rarely seen following radioembolization.

#### Radioembolization in patients with transjugular intra-hepatic portosystemic shunts

Radioembolization can be performed in patients with transjugular intra-hepatic portosystemic shunts (TIPS). An analysis by Memon et al. (55) in patients with TIPS who underwent radioembolization demonstrated new grade 3/4 bilirubin toxicity in 25% of their patients.

### Biliary sequelae

Post-radioembolization biliary complications are potential side effects of radioembolization. The incidence of these complications is less than 10% (33). These may be due to the microembolic effect or radiation-induced injury to the biliary system.

Post-radioembolization biliary complication rates are significantly higher in patients with surgeries/procedures violating the integrity of the ampulla of Vater. ^90^Y radioembolization in the setting of tumor-related biliary obstruction has an acceptable safety profile (36). Biliary complication incidence is also higher in patients who have had polychyemotherapy. Cirrhosis is found to be protective against biliary complications (35).

These patients usually present with pain and can be evaluated with conventional anatomic imaging techniques (33). Incidentally found biliary sequelae on imaging may be seen. Hence, clinical correlation with the imaging findings is necessary (56, 57). Biopsy may be needed in rare cases (34). Following are some biliary complications that have been observed after radioembolization:

#### Radiation cholecystitis

**Figure 4:**
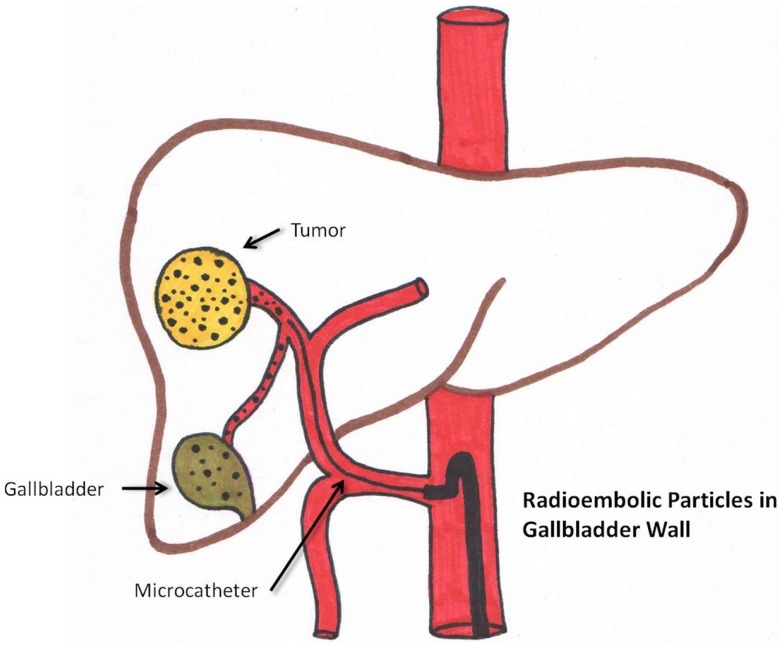
**Schematic representation of aberrant microsphere deposition in the gallbladder wall**.

Radiation cholecystitis may be prevented by identifying the cystic artery. Microsphere injection distal to its origin and coiling can decrease its incidence. (58). This is schematically seen in Figure 4. Cholecystectomy is the treatment of choice.

#### Radiation-induced cholangitis

Fever, jaundice, and right upper quadrant pain may represent radiation-induced cholangitis following radioembolization. Antibiotics may be required.

#### Abscess/bilomas

Abscesses and bilomas are intra-hepatic fluid collections that may form following radioembolization. Bilomas are usually clinically occult and require conservative management. Abscesses may require percutaneous drainage.

#### Other

a) Obstructive jaundice due to biliary strictures
b) Biliary necrosis

### Radiation pneumonitis

**Figure 5:**
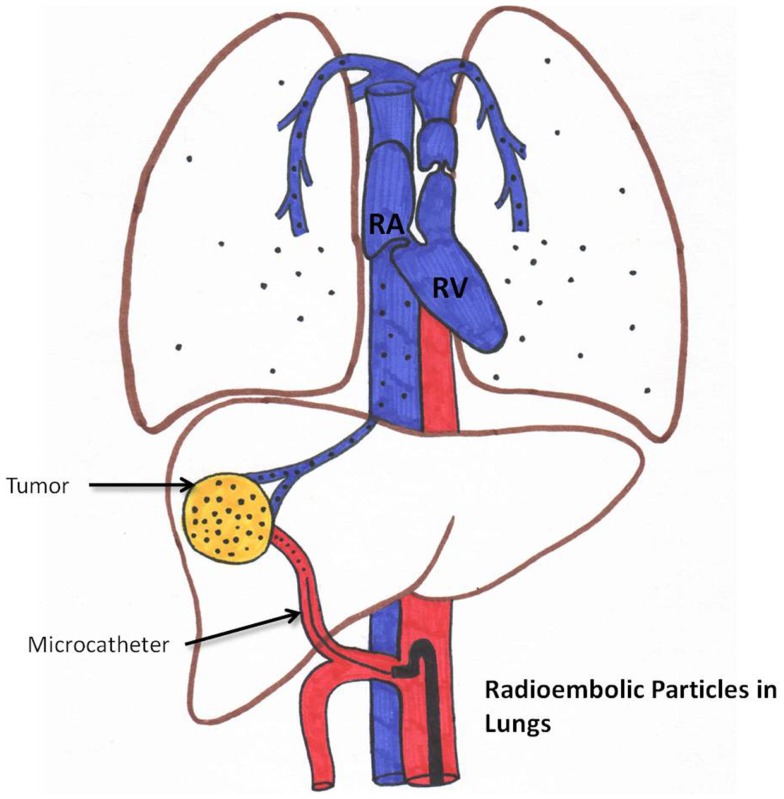
**Schematic representation of aberrant microsphere deposition in the lungs**.

Radiation pneumonitis is very rare (less than 1% if standard dosimetry models are used) (35, 36). This is schematically represented in Figure 5. Please note that if LSF is high, the chance of delivering a high pulmonary dose increases.

Delivery to the lungs of greater than 30 Gray (Gy) in one treatment or a cumulative dose of greater than 50 Gy in multiple treatments is considered a relative contraindication. Planar scintigraphy is usually employed to calculate LSF. Yu et al. described a new method of calculating the mean lung dose for TheraSphere^®^ and SIR-Sphere^®^ radioembolization of liver cancer based on ^99m^Tc-MAA SPECT/CT. According to Yu et al., this method provides a more accurate estimate of radiation risk to the lungs (59). However, this is not routinely performed currently.

A restrictive ventilatory dysfunction following radioembolization has been reported (60). Radiation pneumonitis can be seen as a typical bat-wing appearance on chest CT (35). Data presented by Salem et al. demonstrated a very low incidence of post-radioembolization pulmonary complication (36).

Steroids may play a role in management. Other thoracic complications include atelectasis and/or pleural effusion.

### Gastrointestinal (GI) complications

#### Diarrhea

Diarrhea has been described following radioembolization. This is rarely significant enough to require hospitalization.

#### Gastroenteritis/gastrointestinal ulcera

**Figure 6:**
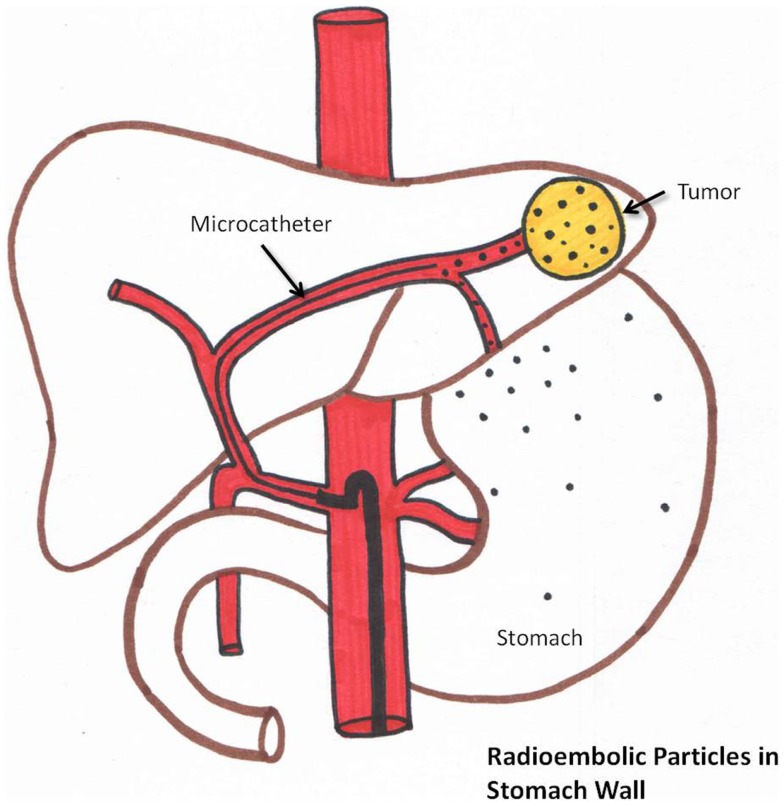
**Schematic representation of aberrant microsphere deposition in the stomach/intestine which can occur due to hepaticogastric communicating arteries**.

Post-radioembolization GI complications occur secondary to hepaticoenteric arterial communications resulting in aberrant microsphere deposition (37). Recognition of these hepaticoenteric arterial communications is essential (38). This is schematically represented in Figure 6. Incidence of GI complications is less than 5% (37–40).

Prophylactic coil embolization of the gastroduodenal and RGA may be considered. The left hepatic angiogram is performed to identify left gastric and inferior esophageal arteries. Delayed angiography of the left hepatic artery with opacification of the coronary vein confirms hepaticoenteric flow. The right hepatic angiogram is required to identify the supraduodenal and retroportal arteries (23).

Prophylactic use of gastric acid suppressive agents (such as proton pump inhibitors) is recommended. If GI ulceration is clinically suspected, endoscopy is recommended to confirm the diagnosis (39, 61).

A recent root cause analysis showed stasis during injection to be the strongest independent risk factor for development of gastroduodenal complications (42). Distal origin of the GDA, young age (*p* = 0.04), and proximal injection of the microspheres were also significant risk factors.

A potential complication of coil embolization of vessels, such as the GDA and RGA, is formation of collateral hepaticoenteric flow. This can result in increased enteric complications on repeat treatments.

### Acute pancreatitis

Acute pancreatitis is a potential but very rare complication of radioembolization (62). Patients present with severe epigastric or periumbilical pain. Serum lipase and amylase levels are usually elevated. Imaging may be helpful to determine other causes of acute pancreatitis. SPECT/CT to detect ^90^Y Bremsstrahlung in the pancreas may be performed. Treatment is conservative.

### Radiation dermatitis

Periumbilical pain may occur due to aberrant microsphere deposition in the anterior abdominal wall via the falciform artery (16, 19, 20). Radiation dermatitis is rare.

Recognition of the falciform artery is essential. Prophylactic embolization of this vessel can be performed if needed to decrease the incidence of radiation dermatitis. Prophylactic topically applied ice prevents complications as it causes vasoconstriction which decreases cutaneous flow (63).

### Lymphopenia

Lymphopenia may be seen after glass microsphere radioembolization. Greater than 25% decrease in lymphocyte count after treatment is seen in the majority of patients (45, 64). However, no opportunistic infections due to the lymphopenia after radioembolization have been reported (45, 64).

## Other Complications

### Thrombocytopenia

A retrospective analysis demonstrated thrombocytopenia as a complication following radioembolization. Splenomegaly can be seen following radioembolization which was shown to be an independent risk factor for development of a low platelet count (65). No significant bleeding diathesis has been reported due to thrombocytopenia following radioembolization.

### Vascular injury

The incidence of vascular injury may be prevented by the following:

a) Knowledge of prior anti-cancer therapy
b) Stopping and resuming “blood thinners” appropriately
c) Reviewing cross-sectional anatomy to determine vascular anatomy such as the furcation of the common femoral artery.

#### Dissection

Newer anti-cancer drugs such as bevacizumab (Avastin) have been shown to make vasculature more friable and prone to injury, increasing the chances of dissection and vascular rupture. Abnormalities in vasculature and hepatic arterial flow in 12/16 (75%) patients who were on anti-cancer therapy has been reported. During angiography, a search for stenoses and abnormal flow should be undertaken (66). Murthy et al. (67) demonstrated a reasonable safety profile of radioembolization with resin microspheres in 10 patients who had been on cetuximab or bevacizumab. Usage of microcatheters and careful wire/catheter manipulation is recommended in patients on or previously exposed to systemic anti-cancer therapy.

Dissection at the site of arteriotomy is rare but possible. A pre-closure common femoral angiogram assists in its diagnosis. However, this may not be routinely performed. The patient may present with a “cold” extremity and stenting/anti-platelet therapy may be required.

#### Bleeding

Hematoma formation at the arteriotomy may be seen in radioembolization (68). Standard protocols mitigating bleeding such as stopping “blood thinners” and making sure the patient’s coagulation profile is within normal limits should be meticulously employed (69). Manual compression may be necessary. Surgical intervention is very rarely required.

If there is suspicion of pseudoaneurysm formation, ultrasound with Doppler may be needed. This is usually treated with ultrasound guided manual compression. Thrombin injection may be performed if necessary. Surgery is rarely necessary.

### Contrast induced nephrotoxicity

It is important to know the patient’s baseline renal function prior to performing transarterial therapies such as radioembolization. Adequate hydration pre- and post-radioembolization and stringent use of iodinated contrast limit contrast induced nephrotoxicity.

### Allergic reaction to iodinated contrast media

Anticipation of possible allergic reactions is essential. Most patients being considered for radioembolization have received prior iodinated contrast media for CT scans. An allergic reaction can range from a minor reaction such as a pruritic rash to an anaphylactoid reaction. If there is a history of prior minor allergic reactions (such as hives) to iodinated contrast, the patient may still received iodinated contrast after receiving an allergy preparation prior to planned transarterial procedure (usually a combination of steroids and anti-histamines).

### Acute chills

Acute chills may occur during treatment, which usually respond to anti-histamines (70).

## Conclusion

Radioembolization is being employed for treatment of various hepatic malignancies. As with any other common therapy, knowledge of potential complications of this therapy is essential. Selecting appropriate patients using a multidisciplinary approach can improve outcomes and decrease complications. Meticulous pre-treatment planning (angiography and ^99m^Tc-MAA scintigraphic imaging) is necessary to minimize side effects of radioembolization. The incidence of complications that may require intervention is low. Grade 3 or higher complications following radioembolization occur in less than 9% of patients. Aberrant microsphere deposition may lead to various complications such as radiation cholecystitis. Surgical therapy may be required in severe cases. As radioembolization is a transarterial procedure, knowledge of general complications associated with transarterial therapies is also important.

## Conflict of Interest Statement

Riad Salem receives research support from Nordion. The other co-authors declare that the research was conducted in the absence of any commercial or financial relationships that could be construed as a potential conflict of interest.
